# Transcriptional and functional characterizations of multiple flagellin genes in spirochetes

**DOI:** 10.1111/mmi.14959

**Published:** 2022-07-18

**Authors:** Kurni Kurniyati, Yunjie Chang, Jun Liu, Chunhao Li

**Affiliations:** ^1^ Department of Oral Craniofacial Molecular Biology, School of Dentistry Virginia Commonwealth University Richmond Virginia USA; ^2^ Microbial Sciences Institute Yale University West Haven Connecticut USA; ^3^ Department of Microbial Pathogenesis Yale School of Medicine New Haven Connecticut USA; ^4^ Department of Microbiology and Immunology, School of Medicine Virginia Commonwealth University Richmond Virginia USA

**Keywords:** motility, flagellin, sigma factors, spirochetes, *Treponema*

## Abstract

The flagellar filament is a helical propeller for bacterial locomotion. In external flagellates, the filaments are mostly homopolymers of a single flagellin protein. By contrast, the flagellar filaments of spirochetes are mostly heteropolymers of multiple flagellin proteins. This report seeks to investigate the role of multiple flagellin proteins using the oral spirochete *Treponema denticola* as a model. First, biochemical and genetic studies uncover that the flagellar filaments of *T. denticola* mainly comprise four proteins, FlaA, FlaB1, FlaB2, and FlaB3, in a defined stoichiometry. Second, transcriptional analyses reveal that the genes encoding these four proteins are regulated by two different transcriptional factors, sigma^28^ and sigma^70^. Third, loss‐of‐function studies demonstrate that each individual flagellin protein contributes to spirochete motility, but none of them is absolutely required. Last, we provide genetic and structural evidence that FlaA forms a “seam”‐like structure around the core and that deletion of individual flagellin protein alters the flagellar homeostasis. Collectively, these results demonstrate that *T. denticola* has evolved a unique mechanism to finely regulate its flagellar filament gene expression and assembly which renders the organelle with the right number, shape, strength, and structure for its distinct motility.

## Graphical abstract

The oral spirochete *Treponema denticola* has periplasmic flagella (PFs) that are mainly composed of three flagellin proteins (FlaB1, FlaB2 and FlaB3) and a sheath protein FlaA. Each protein contributes to the motility but none of them is absolutely required. Structurally, FlaB proteins form a core (14 nm) and FlaA forms a” seam‐like” sheath around the core. The mutant without the sheath has thin PFs (14 nm). By contrast, the wild type has thick PFs (21 nm). This report demonstrates a unique role of multiple flagellin proteins in spirochete flagellation and motility by using an approach of genetics, biochemistry, and cryo‐electron tomography and provides new insights into understanding the complexity and structure of flagellar filament, a helical propeller for bacterial locomotion.

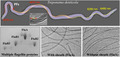

## 1 INTRODUCTION

The bacterial flagellum is a sophisticated nanomachine that consists of three mechanical units: the basal body (motor), the rod‐hook complex (shaft‐joint), and the filament (propeller) (Armitage & Berry, 2020; Chevance & Hughes, 2008; Erhardt et al., 2010). The basal body is imbedded within the cell envelope and works as a reversible rotary motor powered by an inward‐directed electrochemical gradient of protons or sodium ions (Atsumi et al., 1992; Meister et al., 1987; Meister & Berg, 1987). The torque generated by the motor is mechanically transmitted to the flagellar filament, which rotates at hundreds of revolutions per second to propel bacterial locomotion (Berg, 2000). The bacterial flagellar filament has been intensively studied due to its critical role in motility and host‐pathogen interactions, for example, activating host innate immunity through Toll‐like receptors (Hayashi et al., 2001; Yoon et al., 2012). In addition, the flagellar filament has served as an enlightening system for understanding how a polymer composed of a single flagellin protein is assembled and functions as an Archimedean screw to generate mechanical propulsion (Maki‐Yonekura et al., 2010; Samatey et al., 2001; Wang et al., 2017; Yonekura et al., 2003). Assembly of the flagellar filament starts after the flagellar hook is completed, whereby thousands of copies of flagellin proteins are exported through the flagellar‐specific type III secretion system (fT3SS) (Chevance & Hughes, 2008; Erhardt et al., 2010). With the assistance of the cap protein FliD (Al‐Otaibi et al., 2020), an assembling chaperone, the flagellin proteins polymerize to a long helical structure that consists of 11 protofilaments that can adopt both left‐handed (L‐type) and right‐handed (R‐type) conformations due to mechanical forces, such as when the motor switches the sense of rotation (i.e., from counterclockwise to clockwise), allowing bacteria to swim forward or backward or to tumble (Maki‐Yonekura et al., 2010; Pandini et al., 2016; Wang et al., 2017; Yonekura et al., 2003).

Although most bacteria have a single flagellin protein, such as FliC in *Escherichia coli* and *Salmonella typhimurium* (Joys, 1985), and Hag, a homolog of FliC, in *Bacillus subtilis* (LaVallie & Stahl, 1989), some outliers have multiple flagellin proteins. For instance, *Vibrio cholerae* has five flagellin proteins (FlaA, FlaB, FlaC, FlaD, and FlaE) (Klose & Mekalanos, 1998), and both *Helicobacter pylori* and *Campylobacter jejuni* have two flagellin proteins (FlaA and FlaB) (Kostrzynska et al., 1991; Wassenaar et al., 1994). However, in these three bacteria, only FlaA, a homolog of FliC, is a bona fide flagellin protein essential for filament assembly and motility (Echazarreta et al., 2018; Lertsethtakarn et al., 2011; Wassenaar et al., 1994). Flagellin proteins are highly conserved among different bacterial species, as is their quaternary structure. By using cryo‐electron microscopy (cryo‐EM) and helical reconstruction, Galkin et al. reported that the structures of the flagellar filaments from six different bacteria are all composed of 11 protofilaments (Galkin et al., 2008; Wang et al., 2017). In addition to the conservation of amino acid sequences and structures, the regulatory mechanism of flagellin gene expression is also conserved. In most external flagellates, the genes encoding flagellin proteins belong to class III genes in the flagellar regulatory hierarchy, which are finely regulated by sigma^28^ (FliA), a flagellar‐specific alternative transcription activator, and its antagonist, FlgM (Chevance & Hughes, 2008; Lertsethtakarn et al., 2011; Subramanian & Kearns, 2019). Some bacterial flagellin genes, such as *Campylobacter jejuni* and *H. pylori*, are regulated by both sigma^28^ and sigma^54^ (Hendrixson & DiRita, 2003; Prouty et al., 2001; Suerbaum et al., 1993). In addition to transcriptional regulation, the biosynthesis of flagellin proteins in some bacteria can also be regulated at the translational level. For example, CsrA, a small RNA binding protein, negatively regulates the translation of flagellin proteins of *B. subtilis*, *Borrelia burgdorferi*, and several other bacteria (Dugar et al., 2016; Kao et al., 2014; Mukherjee et al., 2011; Romeo & Babitzke, 2018; Sze et al., 2011).

Spirochetes are a unique group of bacteria readily recognized by their flat‐waved or coiled cell morphology and distinct form of corkscrew‐like motility (Charon et al., 1981; Charon et al., 2012; Goldstein et al., 1994) and are responsible for several human diseases, including Lyme disease (*B. burgdorferi*), syphilis (*Treponema pallidum*), and leptospirosis (*Leptospira interrogans*) (Picardeau, 2017; Radolf et al., 2016; Rosa et al., 2005). Unlike external flagellates, spirochetes swim by means of rotating two bundles of periplasmic flagella (PFs) that reside between the outer membrane and cell cylinder (Charon et al., 2012; Li et al., 2000). The number and length of PFs vary from species to species. In general, spirochetal PFs are structurally similar to the flagella of other bacteria as each consists of a basal body‐motor complex, a hook, and a filament. However, the spirochetal flagellar filaments are distinct in terms of their protein composition and structures (Charon et al., 2012; Li et al., 2000; Zhao et al., 2013). For instance, the flagellar filaments of *Brachyspira* and *Treponema* species are composed of three FlaBs (e.g., FlaB1, FlaB2, and FlaB3), homologs of FliC, and a sheath protein, FlaA, that has no sequence similarity to the flagellin proteins (Kurniyati et al., 2017; Li et al., 2000; Li et al., 2000; Li et al., 2008; Norris et al., 1988). The flagellar filament of *Leptospira* is even more complex as it is composed of two FlaA proteins, at least one FlaB protein, and two novel *Leptospira*‐specific flagellar filament proteins, FcpA and FcpB (Gibson et al., 2020; Lambert et al., 2012; Wunder Jr. et al., 2016; Wunder Jr. et al., 2018).

Why spirochetes have evolved multiple flagellar filament proteins and what their roles are with respect to flagellar filament assembly, structure, and bacterial motility are longstanding questions (Charon et al., 2012). We first sought to address this question by using *Brachyspira* (formerly known as *Treponema* and *Serpulina*) *hyodysenteriae*, the first spirochete that could be genetically manipulated. Our results indicate that, while each individual flagellin protein contributes to motility, FlaA appears to be more important than individual FlaBs, for example, deletion of *flaA*, but not *flaBs*, impairs the filament diameter and helicity (Li et al., 2000; Li et al., 2008). A different scenario occurs in *Leptospira biflexa*, in which deletion of *flaB* eliminates filament assembly entirely (Picardeau et al., 2001). In *L. interrogans*, deletion of *flaA* only affects the flagellar filament curvature, but the mutant becomes non‐motile and non‐infectious, highlighting the importance of FlaA in spirochete motility and infectivity (Lambert et al., 2012). Deletions of *fcpA* and *fcpB* abolish the ability of flagella to assume their characteristic supercoiled form and thus dramatically reduce motility and virulence (Wunder Jr. et al., 2016; Wunder Jr. et al., 2018). By integrating high‐resolution cryo‐electron tomography (cryo‐ET) and X‐ray crystallography, Gibson et al presented structural evidence that the *Leptospira* filaments are coated by a highly asymmetric, multi‐component sheath that resides at the filament inner and outer curvatures and defines the supercoiling geometry, a key functional attribute of *Leptospira* PFs (Gibson et al., 2020).

Similar to other spirochetes, *Treponema denticola*, an oral spirochete and keystone pathogen of human periodontitis, possesses flagellar filaments composed of at least one sheath protein FlaA, three flagellin proteins (FlaB1, FlaB2, and FlaB3), and a filament‐associated protein, FlaG (Kurniyati et al., 2017; Kurniyati et al., 2019; Ruby et al., 1997; Seshadri et al., 2004). The three FlaB proteins are modified by a novel 450.2 Da glycan, which is essential for flagellar filament assembly and motility (Kurniyati et al., 2017). The genes encoding these filament proteins were inactivated in our previous report (Kurniyati et al., 2017); however, their roles in flagellar filament assembly, structure, and bacterial motility remain largely unknown. This report aims to fill this knowledge gap by using a multidisciplinary approach of genetics, biochemistry, and cryo‐ET. The results shown here further highlight the complexity and uniqueness of flagellar filaments in spirochetes and provide several new perspectives on what we have learned from the paradigm model organisms of external flagellates such as *E. coli* and *B. subtilis*.

## 2 RESULTS

### 2.1 Measuring the stoichiometry of *T. denticola* flagellar filament proteins

**FIGURE 1:**
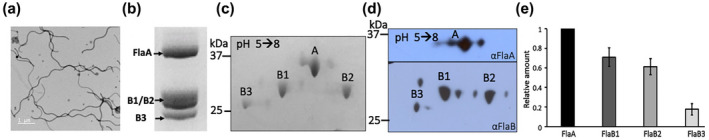
Characterizations of *T. denticola* flagellar filaments. (a) a representative transmission electron microscopic (TEM) image of flagellar filaments isolated from WT. 5 μl of the purified flagellar filaments applied to formvar–carbon copper grids and then stained with 1% uranyl acetate for 1 min (pH 4.2). The samples were subjected to a JEOL JEM‐1400 plus TEM at an acceleration voltage of 120.0 kV. (b) SDS‐PAGE analysis of WT flagellar filaments. (c) 2D gel electrophoresis and (d) immunoblotting analyses of WT flagellar filaments. For the immunoblotting, antibodies against *T. denticola* FlaA (αFlaA) and *T. pallidum* FlaB (αFlaB) were used. (e) the average stoichiometry of the flagellar filament proteins, FlaA, FlaB1, FlaB2, and FlaB3. The densitometry of four flagellar filament proteins was measured from three sets of 2D gels using Bio‐Rad image lab software, and the data is expressed as ratios of FlaB1, FlaB2, and FlaB3 relative to FlaA.

Previous studies indicated that the flagellar filaments of *T. denticola* consist of a sheath protein, FlaA (TDE1712), and three core flagellin proteins, FlaB1 (TDE1477), FlaB2 (TDE1004), and FlaB3 (TDE1475) (Kurniyati et al., 2017; Ruby et al., 1997); however, their stoichiometry in the assembled flagellar filaments remained unknown. To fill this gap, we first purified the flagellar filaments from *T. denticola* ATCC 35405 strain (wild type, WT) to homogeneity (Figure 1a), which were then subjected to SDS‐PAGE for protein separation and quantification. However, the molecular weights (MWs) of the three FlaBs were too similar (30.9–31.6 kDa, Table 1) to be completely separated by SDS‐PAGE (Figure 1b). To overcome this issue, the purified flagellar filaments were subjected to 2D gel electrophoresis followed by Coomassie blue staining (Figure 1c) and immunoblotting probed against FlaA and FlaB antibodies (αFlaA and αFlaB) (Figure 1d). On 2D gels, the four filament proteins were completely separated, and their positions on the gels correlate with the protein isoelectronic points (pIs); for example, FlaB3 (pI 5.3) is more acidic than FlaA (pI 5.39), FlaB1 (pI 5.4), and FlaB2 (pI 6.54) (Figure 1c,d and Table 1). We then measured the densitometry of individual flagellar filament proteins, and the data is expressed as averaged ratios of individual FlaB proteins relative to FlaA (Figure 1e). The stoichiometry of FlaA, FlaB1, FlaB2, and FlaB3 is approximately 1.0:0.7:0.6:0.2 (Figure 1e). We also examined the protein profile of purified PFs by using mass spectrometry. In addition to FlaA, FlaB1, FlaB2, and FlaB3, we also detected a trace amount of other flagellar proteins, such as FlgE (hook protein), FlgK (hook‐associated protein), FliD (cap protein), FliF (MS‐ring protein), and several flagellar rod proteins (e.g., FlgB, FlgC, FlgG, and FlgF) (Table S1). Compared to the flagellin proteins, the abundance of these flagellar proteins is extremely low in the purified PFs. For example, FlgE, the most abundant among these detected minor flagellar proteins, accounts for only 4.2% of FlaB1 (calculated based upon protein molar percentage). Taken together, these results indicate that the flagellar filament of *T. denticola* is a multiple proteinaceous structure that is mainly composed of FlaA, FlaB1, FlaB2, and FlaB3.

**TABLE 1:** Comparison of four flagellar filament proteins of *T. denticola*

Name	Locus tag	Length	Molecular weight (kDa)	Isoelectronic point	Function
FlaA	TDE1712	349 aa	39.3	5.39	Sheath
FlaB1	TDE1477	286 aa	31.3	5.40	Core
FlaB2	TDE1004	286 aa	31.6	6.54	Core
FlaB3	TDE1475	285 aa	30.9	5.30	Core

### 2.2 The flagellar filament genes of *T. denticola* are regulated by different transcriptional factors

**FIGURE 2:**
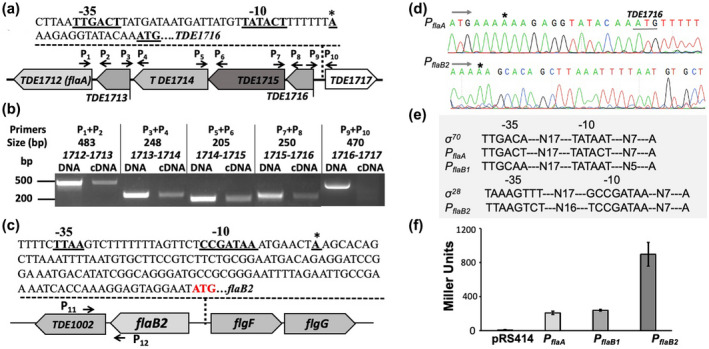
The flagellar filament genes of *T. denticola* are regulated by two transcriptional factors. (a) a diagram showing the upstream region of *flaA* (*TDE1712*) and a promoter‐like sequence identified in the intergenic region between *TDE1716* and *TDE1717*. Arrows represent the relative positions and orientations of RT‐PCR primers; the sequences of these primers are listed in Table 4; asterisk (*) represents the transcriptional start site. (b) co‐RT‐PCR analysis. This experiment was performed as previously documented (Kurniyati et al., 2019). Five pairs of primers that bridge *flaA* and its upstream genes were designed and used for co‐RT‐PCR. For each co‐RT‐PCR reaction, a parallel PCR reaction was performed and used as a positive control. Of note, the pair of P_9_/P_10_ that bridges *TDE1716* and *TDE1717*, two divergently transcribed genes, was used as a negative control to rule out the possibility of DNA contamination in RNA samples. The numbers below the primers are the predicted sizes of PCR products, and the numbers below (e.g., 1712–1713) illustrate the genes that are bridged by each pair of primers (e.g., P_1_/P_2_) for the co‐RT‐PCR analysis. The resultant co‐RT‐PCR and PCR products were detected in 2% agarose gel electrophoresis. (c) a diagram showing the genes adjacent to *flaB2* (*TDE1004*) and its upstream sequence containing a promoter‐like sequence. Red colored ATG is the start codon of *flaB2*. (d) Mapping the transcriptional start sites of *flaA* and *flaB2* genes using 5′‐RLM‐RACE. This experiment was performed using the FirstChoice RLM‐RACE kit (Ambion) according to the manufacturer's protocol. Arrows show the sequencing direction; asterisks (*) are the TSS. (e) Sequence comparison between the *E. coli* sigma^70^ (top panel), sigma^28^ (lower panel) promoters, and the three flagellar filament gene promoters mapped in *T. denticola*, including the promoter sequences upstream of *TDE1716* (*P*_*flaA*_), *TDE1004* (*P*_*flaB2*_), and a previously identified promoter for *flaB1*, *P*_*flaB1*_. (f) Transcriptional analysis of *P*_*flaA*_, *P*_*flaB1*_, and *P*_*flaB2*_ using *lacZ* as a reporter in *E. coli*. For this assay, the three promoters were fused to the promoterless *lacZ* gene in the pRS414 plasmid. The empty vector was used as a negative control. β‐Galactosidase activity was measured and expressed as the average Miller units of triplicate samples from three independent experiments, as previously described. All the primers used here are listed in Table 4.

Our previous study showed that the *flaB1* and *flaB3* genes reside in a large motility gene operon regulated by a sigma^70^‐like promoter, *P*_*flaB1*_ (Kurniyati et al., 2019). However, the regulation of *flaA* and *flaB2* remains unknown. The *flaA* gene (*TDE1712*) is located in a large gene cluster of 11 open reading frames (*orfs*) that starts from *TDE1716* and ends at *TDE1706*. Interestingly, *flaA* is an orphan flagellar gene in this cluster (Seshadri et al., 2004). Co‐reverse trancription‐PCR (Co‐RT‐PCR) analysis using five pairs of primers (P_1_ to P_10_) that bridge *flaA* and its four *orfs* upstream revealed that these five genes are co‐transcribed (Figure 2a,b). There is a 252 bp untranslated region (UTR) upstream of *TDE1716*, the first *orf* of this gene cluster. We first mapped the transcription start site (TSS) 14 bp from the start codon of *TDE1716* (Figure 2d), using RNA ligase‐mediated rapid amplification of cDNA ends (RLM‐RACE) analysis. A search of this region for promoter‐like sequences revealed that the −10 (TTGACT) and −35 regions (TATACT) from the TSS contain a consensus sequence of sigma^70^ promoter (Figure 2a,e). The identified promoter is designated *P*_*flaA*_.

Upstream of *flaB2* (*TDE1004*) is *flgF*, a gene encoding the flagellar rod, but this gene is in an opposite orientation (Figure 2c). Downstream of *flaB2* is *TDE1002*, a gene encoding a putative membrane protein. Co‐RT‐PCR analysis showed that *TDE1002* and *TDE1004* are co‐transcribed (Figure S1). We mapped the TSS, which is 124 bp from the start codon of *flaB2*, using RLM‐RACE (Figure 2c). We then searched the upstream region of TSS and found that the −10 (CCGATAA) and −35 (TTAA) regions from the TSS contain a sigma^28^ promoter consensus sequence (Figure 2c). The identified promoter was named *P*_*flaB2*_. We then carried out transcriptional reporter assays using *lacZ* to determine if the identified three promoters (*P*_*flaA*_, *P*_*flaB1*_, and *P*_*flaB2*_) are functional. The result showed that all three promoters drive the expression of *lacZ* in *E. coli*, with *P*_*flaB2*_ the strongest (899 ± 140 Miller units), *P*_*flaA*_ of medium strength (665 ± 94 Miller units), and *P*_*flaB1*_ the weakest (243 ± 11 Miller units) (Figure 2f). Collectively, these results indicate that the flagellar filament genes of *T. denticola* are differently regulated, whereby *flaB2* is controlled by sigma^28^, and the other genes are regulated by sigma^70^.

### 2.3 The flagellar filament genes of *T. denticola* have different expression patterns

**FIGURE 3:**
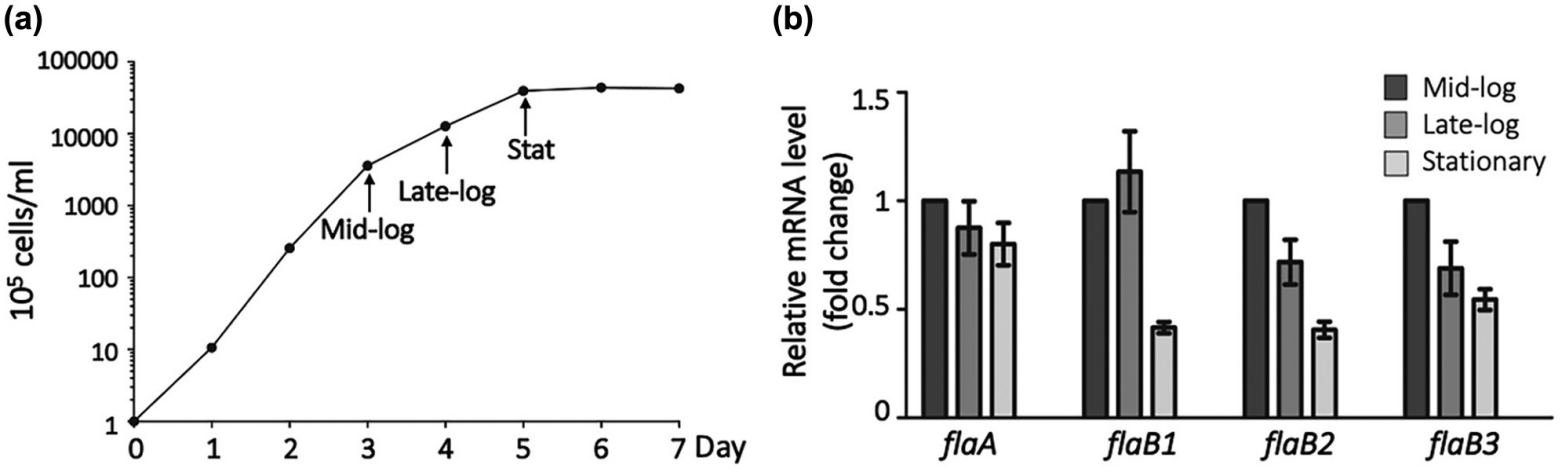
Measuring the expression of four flagellar filament genes during *T. denticola* growth. (a) the growth curve of *T. denticola*. For this experiment, *T. denticola* wild‐type ATCC 35405 (WT) was grown in TYGVS medium and enumerated every 24 h using a Petroff‐Hausser counting chamber. (b) the expressional level of *flaA*, *flaB1*, *flaB2*, and *flaB3* at different growth phases. *T. denticola* cultures were collected at the mid‐log, late‐log, and stationary phase as indicated in (a) and subjected to RNA isolation followed by qRT‐PCR, which was performed using iQ SYBR green supermix and a MyiQ thermal cycler, as previously described (Kurniyati et al., 2017; Kurniyati et al., 2019). The molecular chaperone DnaK gene (*dnaK*, *TDE0628*) was used as an internal control to normalize the mRNA level of each individual genes. The results were expressed as mRNA level (ΔΔ*C*_*T*_) of the mid‐phase relative to the late‐log phase or stationary phase. Triplicates were included for each experiment. The data shown here is the average of three independent experiments. The primers for qRT‐PCR are listed in Table 4.

Given that the four flagellar filament genes of *T. denticola* are regulated by two different promoters, we speculated that they may have different expression dynamics during growth. To determine if this is the case, we isolated the RNA samples from *T. denticola* cells harvested at days 3 (middle log), 4 (late log), and 5 (stationary) (Figure 3a) and then measured the expression levels of four flagellar filament genes relative to that of *dnaK* (*TDE0628*), a housekeeping gene of *T. denticola*, using quantitative reverse transcription‐PCR (qRT‐PCR), as previously documented (Kurniyati et al., 2019). The *flaA* gene was constantly expressed and not affected by the growth phase, whereas the three *flaB* genes were actively expressed at the middle log phase, and then *flaB2* and *flaB3* started to decline at the late log phase, as did *flaB1* at the stationary phase (Figure 3), suggesting that the expression of the three flagellin genes is affected by the growth phase of *T. denticola*.

### 2.4 Assessing the role of individual flagellar filament genes in the motility of *T. denticola*

**FIGURE 4:**
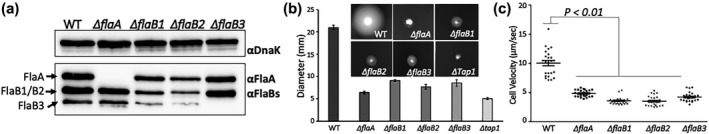
Characterizations of four flagellar filament gene deletion mutants. (a) Whole‐cell lysate immunoblotting analysis of WT and the four flagellar filament gene deletion mutants. The blots were probed with antibodies against *T. denticola* DnaK (αDnaK), FlaA (αFlaA), and *T. pallidum* FlaB (αFlaB), respectively. DnaK was used as a loading control. (b) Swimming plate assay. This assay was carried out on 0.35% agarose plates containing the TYGVS medium diluted 1:1 with PBS. The plates were incubated anaerobically at 37°C for 3 days to allow the cells to swim out. *Δtap1*, a previously constructed non‐motile mutant (Limberger et al., 1999), was used as a control to determine the initial inoculum sizes. The sizes of swimming rings from 10 different plates were measured and averaged. (c) Cell tracking analysis. *T. denticola* cells were tracked in the presence of 1% methylcellulose, as previously described (Kurniyati et al., 2017). The results are expressed as the mean of μm/s ± standard errors of mean (SEM). The data were analyzed by one‐way ANOVA followed by Tukey's multiple comparison at *p* < .01.

We previously *in‐frame* replaced four individual flagellar filament genes with an erythromycin resistance marker (*ermB*) (Kurniyati et al., 2017); the resulting mutants are designated as: *ΔflaA*, *ΔflaB1*, *ΔflaB2*, and *ΔflaB3*. Western‐blot analyses using whole‐cell lysates showed that the cognate gene products were abolished in these mutants as expected, for example, FlaA was absent in *ΔflaA*, and FlaB3 was abolished in *ΔflaB3* (Figure 4a). These four mutants were less motile than WT under dark‐field microscopy (Videos [Link], [Link]), suggesting that motility is impaired in these mutants. We then quantitatively assessed the impact of individual flagellar filament genes on *T. denticola* motility by using swimming plate assays and a computer‐based bacterial tracking system, as previously described (Kurniyati et al., 2019). Swimming plate assays showed that the swimming rings formed by the four mutants are significantly (*p* < .01) smaller than those of WT but larger than those of *Δtap1*, a non‐motile mutant previously constructed (Limberger et al., 1999) (Figure 4b). By using the bacterial tracking system, we measured the cell velocity (μm/sec, *n* = 24 cells) of WT and the four mutants. The averaged cell velocity of WT is 10.04 ± 0.5, approximately 2‐3‐fold faster than that of *ΔflaA* (4.8 ± 0.1), *ΔflaB1* (3.6 ± 0.1), *ΔflaB2* (3.5 ± 0.2), and *ΔflaB3 (*4.2 ± 0.2) mutants (Figure 4c). Notably, there is no significant difference (*p* > .01) between individual mutants in both swimming plate assays and tracking analyses. Collectively, these results indicate that all four flagellar filament genes contribute to motility, but none of them is absolutely required.

### 2.5 FlaA forms a “seam”‐like sheath structure

**FIGURE 5:**
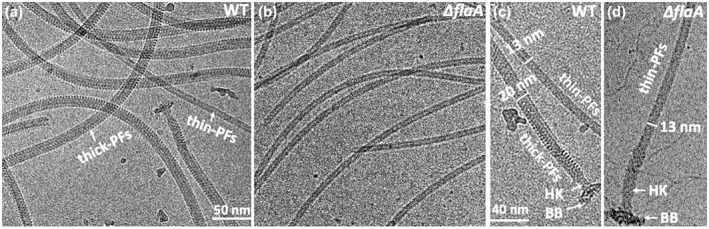
Cryo‐EM analysis of isolated PFs. (a,c) representative TEM images of WT PFs. (b,d) representative cryo‐electron microscopic images of PFs isolated from the *ΔflaA* mutant. The diameter of thick‐PFs with sheath is about 20 nm and of thin‐PFs without sheath about 13 nm. PFs: Periplasmic flagella; BB: Basal body; HK: Hook.

It has been proposed that FlaB proteins form the core that is sheathed by FlaA (Charon et al., 2012; Li et al., 2000); however, there is lack of solid genetic and physical evidence to support this proposition. To fill this gap, we first measured the flagellar filament diameters in *T. denticola* cells using cryo‐ET. Both WT and three *flaB* mutants have two types of PFs, referred to as “thick‐PFs” and “thin‐PFs”. The thick‐PFs are the predominant species and average 20 nm in diameter; the thin‐PFs constitute a minority and average 13.5 nm in diameter (Table 2). By contrast, the *ΔflaA* mutant has only thin‐PFs (13.9 ± 1.5 nm). We next isolated the PFs from WT and *ΔflaA* and examined their ultrastructure using cryo‐EM. As in the whole‐cell cryo‐ET analysis, the majority (>95%) of PFs in WT are thick (average 20 nm in diameter) (Figure 5a). By contrast, the PFs of *ΔflaA* are uniformly thin (average 13.5 nm in diameter) (Figure 5b). Interestingly, the WT PFs have a rough surface which is covered by a “seam”‐like structure (Figure 5a). In intact PFs, this “seam”‐like structure starts from the filament tip, extends to the entire filament, and stops at the interface between the filament and hook (Figure 5c). By contrast, the PFs of *ΔflaA* are uniformly thin and smooth and have no such “seam”‐like structures observed (Figure 5b,d). We also examined the PFs isolated from the three *flaB* mutants. They are morphologically indistinguishable from WT (Figure S2). Based on these observations, we propose that FlaA, as a sheath protein, forms the “seam”‐like structure around the core formed by three FlaB proteins.

**TABLE 2:** The length of PFs in WT and the four flagellar filament mutants

	WT*n* = 12 cells	*ΔflaAn* = 6 cells	*ΔflaB1n* = 7 cells	*ΔflaB2n* = 8 cells	*ΔflaB3n* = 4 cells
Thick PFs	19.5 ± 1.7 nm	None	19.9 ± 2.1 nm	21.1 ± 1.4 nm	20.6 ± 2.0 nm
	(*n* = 30)		(*n* = 30)	(*n* = 30)	(*n* = 30)
Thin PFs	14.2 ± 1.4 nm	13.9 ± 1.5 nm	13.0 ± 1.1 nm	13.6 ± 1.6 nm	13.4 ± 1.6 nm
	(*n* ^a^ = 30)	(*n* = 30)	(*n* = 30)	(*n* = 30)	(*n* = 30)
PF length	6.3 ± 0.9 μm (*n* ^a^, ^b^ = 29)	7.5 ± 0.8 μm (*n* = 12)	4.2 ± 0.8 μm (*n* = 12)	3.7 ± 1.6 μm (*n* = 11)	4.5 ± 0.6 μm (*n* = 8)
	4. 0 ± 1.5 μm (*n* = 27)	4.7 ± 1.3 μm (*n* = 12)	2.7 ± 0.9 μm (*n* = 14)	2.5 ± 0.9 μm (*n* = 14)	3.0 ± 0.8 μm (*n* = 8)

^a^ Measurements.

^b^ Number of PFs.

### 2.6 FlaA controls the helicity of PFs

**FIGURE 6:**
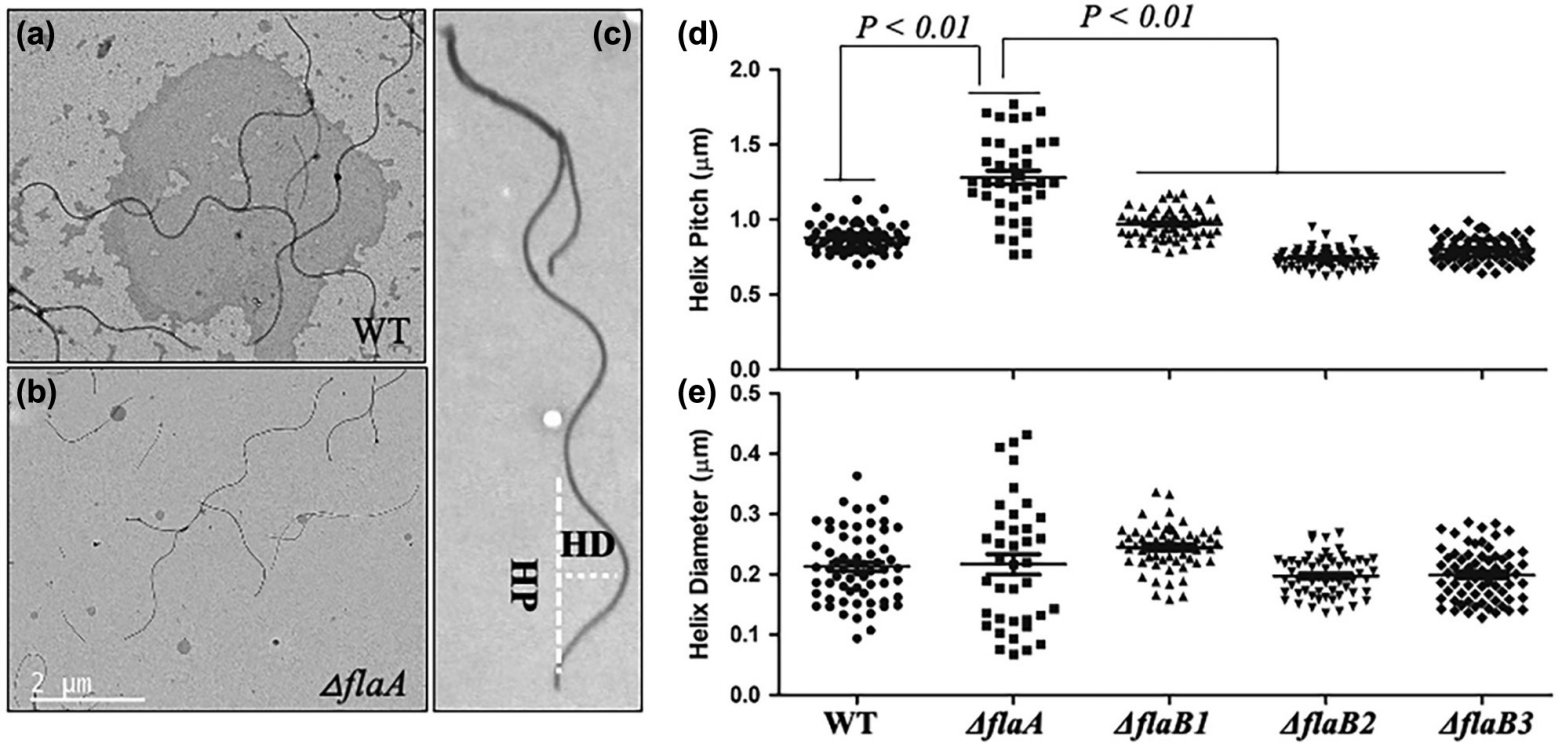
Measuring the helicity of PFs. (a,b) representative TEM images of PFs isolated from WT and the *ΔflaA* mutant. (c) a TEM image of intact WT PFs showing helix pitch (HP) and helix diameter (HD). (d) Bar graph of helix pitch and (e) Bar graph of helix diameter of WT and the four flagellar filament gene deletion mutants. The results are expressed as the mean of μm/s ± standard errors of mean (SEM). The data were analyzed by one‐way ANOVA followed by Tukey's multiple comparison at *p* < .01.

Bacterial flagella are helical structures whose helicity can be measured by helix pitches and helix diameters (Charon et al., 2012, Li et al., 2000). We examined the isolated PFs using transmission electron microscopy (TEM). The PFs isolated from *ΔflaA* appear more fragile (more broken PFs observed) and less helical than those of WT and the three *flaB* mutants (Figures 6a,b and S3). To further confirm this observation, we measured the helix pitch (HP) and helix diameter (HD) of isolated PFs from WT and the four mutants. Deletion of individual *flaB* genes has a neglectable impact on flagellar helicity, for example, the PFs isolated from WT and the three *flaB* mutants have similar sizes of HP and HD (Figure 6d,e). Like the three *flaB* mutants, deletion of *flaA* has no obvious impact on HD (Figure 6e) but significantly increases the size of HP (Figure 6d), for example, the average size of HP in the WT PFs is 0.88 ± 0.01 μm (*n* = 63 PFs), which increases to 1.28 ± 0.04 μm (*n* = 38 PFs) in *ΔflaA*. These results demonstrate that the PFs of *ΔflaA* are less helical than those of WT and the three *flaB* mutants, indicating that among these four flagellar filament proteins, FlaA is the major determinant of flagellar helicity.

### 2.7 Characterizations of individual *flaB* mutants

**FIGURE 7:**
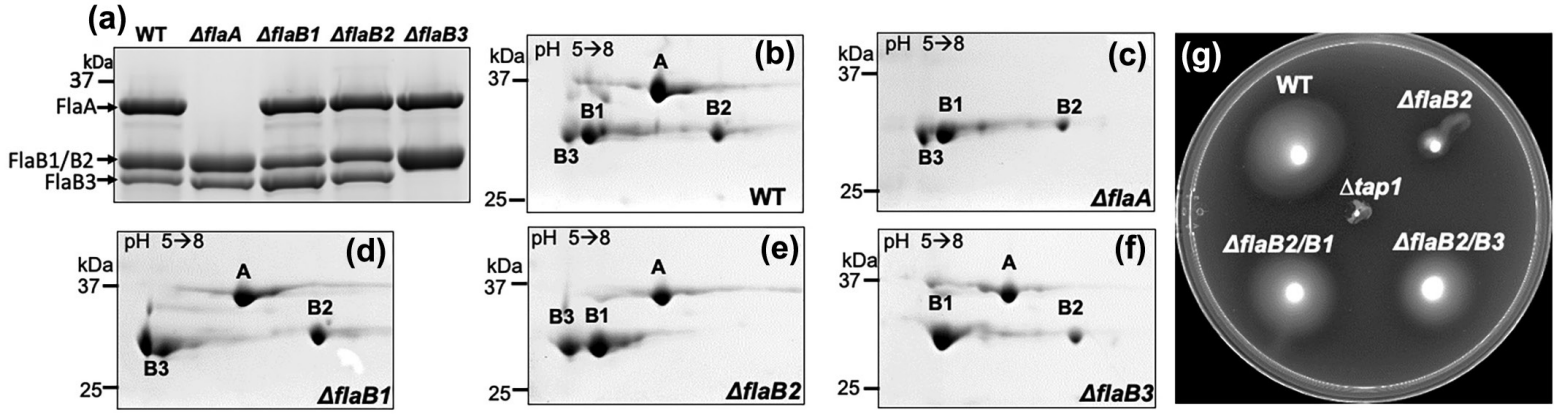
The three FlaB proteins are functionally interchangeable. (a) SDS‐PAGE analysis of PFs isolated from WT and the flagellar filament gene deletion mutants. (b–f) 2D gel analysis of PFs isolated from WT and the four flagellar filament gene deletion mutants. For the SDS‐PAGE and 2D gel analyses, the same amounts of isolated PFs from WT and the four mutants were loaded. (g) Swimming plate assay. This assay is conducted as described in Figure 4b. *ΔflaB2/B1* and *ΔflaB2/B3* are two mutants in which the *flaB2* gene was in‐frame replaced with either *flaB1* or *flaB3*. These two mutants were confirmed by PCR and western‐blot analyses.

In the above study, we measured the stoichiometry of four flagellar filament proteins in the WT PFs (Figure 1). Interestingly, SDS‐PAGE analysis of isolated PFs suggested that deletions of individual *flaB* genes changed the stoichiometry of three FlaB proteins (Figure 7a). For example, deletion of *flaB1* significantly increased the level of FlaB3. To confirm this observation, 2D gel analysis shown in Figure 1c was conducted to measure the stoichiometry of flagellar filaments in the four mutants (Table 3). Deletion of *flaA* had no significant impact on the stoichiometry of three FlaBs (Figure 7c), but there was a trend whereby deletion of one *flaB* increased the level of the other FlaB proteins in the isolated PFs (Figure 7d–f). For example, deletion of *flaB1* increased the ratio of FlaB3 to FlaA from 0.2:1 to 1:1.12, an approximately 5‐fold increase; deletion of *flaB2* increased the ratio of FlaB1 and FlaB3 to FlaA from 0.7:0.2:1 to 1.0:0.4:1. These results suggest that the three FlaB proteins can reciprocally rescue each other. If so, we would expect the three FlaB proteins to be functionally interchangeable. To test this hypothesis, we *in‐frame* replaced the *flaB2* gene with either *flaB1* or *flaB3* via allelic exchange. The resultant two mutants (*ΔflaB2/B1* and *ΔflaB2/B3*) were confirmed by PCR and western‐blot analysis (Figure S4). Swimming plate assays revealed that the gene replacement had no obvious impact on the motility of *T. denticola* (Figure 7g) and tracking analysis showed that the velocities (μm/sec, *n* = 18 cells) of *ΔflaB2/B1* (12.4.0 ± 0.49) and *ΔflaB2/B3* (10.3 ± 0.48) are slightly less than that of WT (14.0 ± 0.56) but the difference is not statistically significant. Collectively, these results indicate that the three FlaB proteins are, at least in part, functionally interchangeable which allows them to rescue each other.

**TABLE 3:** The stoichiometry of four flagellar filament proteins in the PFs of WT and four mutants

	FlaA	FlaB1	FlaB2	FlaB3
WT	1.00	0.71 ± 0.09	0.61 ± 0.08	0.18 ± 0.06
*ΔflaB1*	1.00		0.5 ± 0.05	0.94 ± 0.26
*ΔflaB2*	1.00	1.04 ± 0.08		0.38 ± 0.2
*ΔflaB3*	1.00	1.12 ± 0.2	0.36 ± 0.05	

*Note*: The stoichiometry was determined based upon the densitometry of individual proteins in three to five separate 2‐D gels, and the data is expressed as averaged ratios of individual FlaB proteins relative to FlaA.

### 2.8 Individual flagellar filament proteins are important for the homeostasis of PFs

**FIGURE 8:**
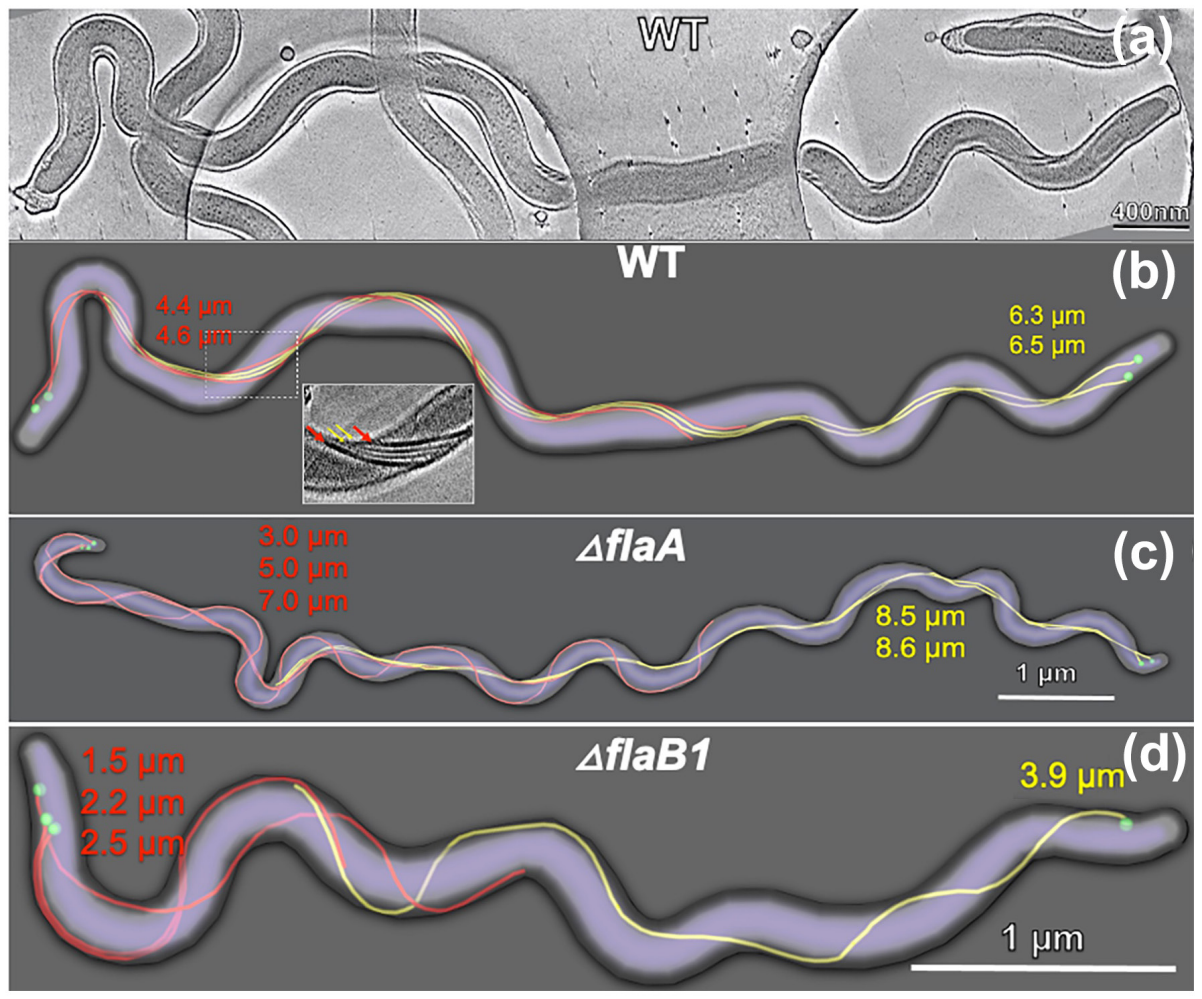
Whole‐cell tomography analysis of spirochetes and *T. denticola* flagellar filament deletion mutants. (a,b) a cryo‐ET image and surface view of a *T. denticola* WT cell. (c,d) surface views of two representative mutant cells of *ΔflaA* and *ΔflaB1* mutants, respectively. The PFs originating from two cell poles are labeled with different colors.

The number and length of PFs vary among different spirochetes. For example, *B. burgdorferi* has 7–12 PFs at each cell pole which form two long ribbon‐like structures that wrap around the cell cylinder in a right‐handed sense (Sze et al., 2011; Zhang et al., 2020). By contrast, *Leptospira* species have only one short flagellum at each pole (Goldstein & Charon, 1988; Raddi et al., 2012). Whole‐cell cryo‐ET analysis showed that *T. denticola* has two long helical PFs that arise from two cell poles and extend toward the opposite ends. As in *B. burgdorferi*, those PFs form a tightly packed ribbon‐like structure and overlap at the central region of the cells (Charon et al., 2009; Zhang et al., 2020). Interestingly, we observed that the two bundles of PFs in *T. denticola* are asymmetric in terms of length (Figure 8a,b); for example, the PFs from one end of the cell (6.3 μm, *n* = 12 cells and 29 PFs) are longer than those from the opposite end (4.0 μm, *n* = 12 cells and 27 PFs), but the PFs from the same cell pole typically have similar lengths. One conceivable explanation is that the cell end with long PFs is the old cell pole, and the one with short PFs is the nascent cell pole. Cryo‐ET analyses of multiple WT cells showed that they all have a similar pattern in terms of flagellar number, length, location, and configuration. We refer to this phenomenon as homeostasis. Intriguingly, this homeostasis is disrupted in the four flagellar mutants (Figure 8, Tables 2 and S2). Compared to the WT, these mutants are more diverse in terms of flagellar number, length, and distribution. Some mutant cells have asymmetrical numbers of PFs at the two cell poles, for example, one pole with multiple PFs and the other end with only one. In addition, the PFs in these mutants are more diverse with respect to length, for example, most PFs have normal lengths (ranging from 3 to 6 μm), but occasionally short (<2 μm) and long PFs (>12 μm) were also observed. More strikingly, the PFs in these mutants are unable to form a tightly packed ribbon‐like structure as observed in WT (Figure 8). These results indicate that the presence of individual filament proteins is essential for spirochetes to maintain flagellar homeostasis (i.e., number, length, and configuration).

## 3 DISCUSSION

### 3.1 Complexity of multiple flagellar filament gene regulation

In this report, we first demonstrate that the four flagellar filament genes are regulated by two different transcriptional factors, sigma^70^ and sigma^28^. Consistent with this observation, both sigma^70^ (RpoD, TDE1346) and sigma^28^ (FliA, TDE2683) homologs are found in the genome of *T. denticola* (Seshadri et al., 2004). In addition, we recently identified an FlgM homolog (TDE0201) in *T. denticola*. Our preliminary data revealed that TDE2683 binds to the sigma^28^ promoter (e.g., *P*_*flaB2*_) and is essential for the expression of the *flaB2* gene and that TDE0201 functions as an antagonist of TDE2683. In addition to sigma^70^, sigma^28^, and FlgM, we recently found a CsrA homolog (TDE2355) in a flagellar gene cluster of *T. denticola* (*flgN‐flgK‐flgL‐fliW‐csrA*) and its binding sites near the Shine‐Dalgarno (SD) sequences of *flaB1* and *flaB2*, suggesting that CsrA is also involved in the regulation of flagellin synthesis of *T. denticola*, most likely at the translational level, as reported in *B. subtilis*, *B. burgdorferi*, and several other flagellated bacteria (Dugar et al., 2016; Kao et al., 2014; Mukherjee et al., 2011; Sze et al., 2011). Collectively, these results demonstrate that *T. denticola* has evolved a complex, multi‐layer regulatory network to finely orchestrate its flagellar filament protein synthesis and assembly. A similar regulatory mechanism has also been reported in other bacteria that have multiple flagellin proteins, such as *B. hyodysenteriae* (Li et al., 2010), *V. cholerae* (Klose & Mekalanos, 1998; Prouty et al., 2001), and *C. jejuni* (Hendrixson & DiRita, 2003; Lertsethtakarn et al., 2011). Like *T. denticola*, the flagellar filaments of *B. hyodysenteriae* also comprise one FlaA and three FlaB proteins, with the genes encoding FlaA and FlaB3 regulated by sigma^70^ and those encoding FlaB1 and FlaB2 controlled by sigma^28^ (Li et al., 2000; Li et al., 2008; Li et al., 2010). *V. cholerae* employs sigma^54^ to control its major flagellin (FlaA) gene expression and sigma^28^ for the transcription of four minor flagellins (Prouty et al., 2001). The flagellar filament of *C. jejuni* is composed of a major (FlaA) and minor (FlaB) flagellin regulated by sigma^28^ and sigma^54^, respectively (Guerry et al., 1991; Hendrixson et al., 2001). In addition, the *flaA* mRNA is translationally regulated by CsrA (Radomska et al., 2016). Notably, the FlaA proteins of *V. cholerae* and *C. jejuni* are homologs of FliC and have no sequence similarity to the spirochete FlaA proteins.

Compared to peritrichous flagellates, polar flagellates with multiple flagellins have evolved more complex regulatory mechanisms to modulate their flagellin gene expression and flagellar assembly (Charon et al., 2012; Josenhans et al., 2002; Josenhans et al., 2002; Kazmierczak & Hendrixson, 2013; Khan et al., 2020; Niehus et al., 2002). This complexity allows them to exquisitely orchestrate the levels of individual proteins for the assembly of flagellar filaments, a sophisticated macromolecule composed of multiple proteinaceous units. For example, the four flagellar filament proteins of *T. denticola* have a defined stoichiometry of 1:0.7:0.6:0.2 (Figure 1e). Thus, it needs a complex regulatory system to finely harness the level of individual proteins. The other advantage is that this complexity may allow these bacteria to dynamically change their flagellar filament composition and physical features (e.g., length and strength) by altering the expression level of individual flagellins and their ratio in response to environmental stimuli and a change of hosts. For example, *H. pylori* can alter its flagellin gene expression in response to changes in growth phase, pH, and quorum sensing (Loh et al., 2004; Merrell et al., 2003; Thompson et al., 2003). *C. jejuni* has a major flagellin FlaA and minor flagellin FlaB; however, there is a shift in expression from *flaA* to *flaB* during invasion (Wassenaar et al., 1994). Similarly, *T. denticola* can also alter the level of individual flagellar filament proteins in response to environmental changes, for example, the levels of FlaA and FlaB2 increased considerably when *T. denticola* formed dual‐species biofilms with the oral bacterial pathogen *Porphyromonas gingivalis* (Zhu et al., 2013).

### 3.2 Unique FlaA sheath proteins in spirochetes

It is of note that flagellar sheaths are also found in other Gram‐negative bacteria, such as *H. pylori*, *C. jejuni*, and *V. cholera* (Chu et al., 2020; Lertsethtakarn et al., 2011); however, these sheaths are fundamentally different from the flagellar sheath of spirochetes. Electron microscopy analyses uncovered that the flagellar sheaths in those non‐spirochetal bacteria are an extension of the cell outer membrane (OM) that encases the flagella (Chu et al., 2020). The sheath found in these bacteria is loosely attached to the flagella and can be easily removed by treatment with detergents or even by physical methods such as washing (Sjoblad et al., 1983; Yang et al., 1977). Little is known about the composition of these flagellar sheaths and their roles. Biochemical analyses of the flagellar sheaths from a few bacterial species suggest that they contain lipopolysaccharide, phospholipids, and OM proteins (Fuerst & Perry, 1988); however, these studies are inconclusive, and some are even controversial. Various functions for the flagellar sheath have been proposed, including preventing disassociation of flagellin subunits in the presence of gastric acid, avoiding activation of the host innate immune response by flagellin, adherence to host cells, and protecting the bacterium from bacteriophages (Carlsohn et al., 2006; Chu et al., 2020; Jones et al., 1997; Zhu et al., 2018). However, the experimental evidence that supports these speculations is limited.

FlaA sheath proteins are unique in terms of their sequence, function, and structure. First, FlaA proteins have thus far been found only in the phylum of spirochetes and do not share sequence similarity with other flagellar proteins, including flagellins, such as *E. coli* FliC and the FlaB proteins of spirochetes. In addition, FlaA proteins are not exported through fT3SS as other flagellar proteins are. Instead, they are likely exported to the periplasmic space by the type II secretion pathway, as their N‐terminal amino sequences are cleaved, and a typical peptidase I cleavage site is present near the N termini (Li et al., 2000; Norris et al., 1988). Second, accumulating evidence suggests that the roles of FlaA proteins in spirochetes are varied. For instance, the genome of *L. interrogans* encodes two *flaA* genes, *flaA1* and *flaA2*. A loss of function study revealed that a deletion mutant of *flaA1* retains normal flagella, morphology, and virulence, albeit with reduced motility, whereas a deletion mutant of *flaA2* is aflagellated, non‐motile, and defective in causing disease in animal models of acute infection, highlighting a critical role of FlaA2 in flagellar synthesis, motility, and virulence (Lambert et al., 2012). Our previous studies indicated that deletion of *flaA* impairs flagellar the filament diameters, helicity, and motility of *B. hyodysenteriae* (Li et al., 2000). Herein, a similar phenotype is observed in the *flaA* mutant of *T. denticola* (Figures 4, 5, 6). By contrast, deletion of *flaA* in *B. burgdorferi* has no obvious impact on flagellar morphology, assembly, or motility. Finally, emerging evidence suggests that FlaA proteins form a different structure. It has been proposed that FlaA proteins form a sheath around the filament core (Charon et al., 1992; Charon et al., 2012; Li et al., 2000; Li et al., 2000). This model was initially built upon early immuno‐electron microscopy studies using FlaA antibodies, whereby FlaA proteins are pinpointed at the surface of flagellar filaments, and later substantiated by our previous studies of using *B. hyodysenteriae* as a genetic model (Cockayne et al., 1987; Li et al., 2000; Trueba et al., 1992). In these studies, FlaA was found to form a hollow tubular‐like structure that confers extra thickness and strength upon the flagellar filament. The data shown in this report further corroborates this model. In addition, more structural details are revealed, for example, the sheath of FlaA is a “seam”‐like structure that encases the filaments (Figure 5). Interestingly, a recent structural study revealed that the FlaA proteins of *Leptospira* bind to FcpA and FcpB and form an asymmetric, lopsided architecture rather than the tubular‐like sheath observed in *B. hyodysenteriae* and *T. denticola* (Gibson et al., 2020). *B. burgdorferi* has a minor FlaA protein (Ge et al., 1998; Ge & Charon, 1997). Our recent immuno‐fluorescence microscopy study revealed that FlaA does not assembled around the filament core. Instead, it is located at the interface between the flagellar hook and filament (Li et al., unpublished data). Its role and structure in *B. burgdorferi* remain unknown. From these four exemplified spirochetes, we conclude that the role and structure of FlaA proteins vary from species to species despite their amino acid sequences being conserved among spirochetes.

### 3.3 Overlapping role of three FlaB proteins in *T. denticola*

Except for *B. burgdorferi*, most spirochetes have multiple flagellin proteins that are highly conserved with respect to their amino acid sequences (Charon et al., 2012; Li et al., 2008; Norris et al., 1988; Wunder et al., 2016). For example, the three FlaB proteins of *T. denticola* share at least 70% sequence identity and 80% sequence similarity. A similar scenario stands for the FlaB proteins in other spirochetes. It remains unclear why multiple FlaB proteins exist or whether these flagellins adopt distinct structural or functional roles. The results shown in this report provide some answers to these questions. First, the loss‐of‐function study reveals that even though each individual FlaB protein contributes to the motility of *T. denticola*, none is absolutely required, for example, the three *flaB* mutants are still able to assemble flagellar filaments (Figures 7, S2, and S3), and their motility is only partially impaired (Figure 4). In addition, our recent studies disclosed that double deletion mutants of *flaB1flaB2*, *flaB1flaB3*, and *flaB2flaB3* still retain partial motility (Figure S5). Second, 2D gel analyses reveal that deletion of one *flaB* leads to augmentation of other flagellins in the assembled flagellar filaments (Figure 7). Last, genetic studies indicate that substitution of *flaB2* with either *flaB1* or *flaB3* has no obvious impact on the motility of *T. denticola* (Figures 7g and S4). In contrast to those spirochetes that have multiple flagellin isoforms, *B. burgdorferi* has only one FlaB protein, which is essential for flagellar filament assembly and motility; for example, deletion of *flaB* leads to a mutant that has no flagellar filament and is thus non‐motile (Motaleb et al., 2000). Taken together, these findings indicate that the multiple FlaB isoforms are, at least in part, functionally interchangeable which makes *T. denticola*, and perhaps other spirochetes as well, more resilient to mutations. As mentioned in our introduction, bacterial flagellar filaments are structurally conserved and typically composed of 11 protofilaments. It is also possible that these multiple flagellin isoforms render spirochete flagellar filaments a different structure. We are currently attempting to delineate the structural details of spirochetal flagellar filaments using the mutants constructed in this report.

In summary, this report further highlights the complexity and uniqueness of spirochete flagellar filaments and provides several new perspectives to understanding of the regulatory mechanism of multiple flagellar filament proteinaceous units as well as their roles in spirochete flagellar filament assembly, structure, and locomotion. These perspectives, alongside the materials (e.g., mutants and the PFs isolated from these mutants) and methodologies (e.g., whole‐cell cryo‐ET) generated in this report, pave the way for future study, for example, elucidating the structural details and assembly mechanism of flagellar filaments by using single‐particle cryo‐EM and cryo‐ET.

## 4 EXPERIMENTAL PROCEDURES

### 4.1 Bacterial strains, culture conditions, and oligonucleotide primers

*T. denticola* ATCC 35405 (wild‐type) and mutant strains were grown in tryptone‐yeast extract‐gelatin‐volatile fatty acids‐serum (TYGVS) medium at 37°C in an anaerobic chamber in the presence of 85% nitrogen, 5% carbon dioxide, and 5% hydrogen, as previously documented (Kurniyati et al., 2017; Seshadri et al., 2004). *T. denticola* isogenic mutants were grown with appropriate antibiotic for selective pressure as needed: erythromycin (50 μg/ml) and gentamicin (20 μg/ml). *Escherichia coli* DH5α strain (New England Biolabs, Ipswich, MA) was used for DNA cloning. The *E. coli* strains were cultivated in lysogeny broth (LB) supplemented with appropriate concentrations of antibiotics for selective pressure as needed: ampicillin (100 μg/ml). The oligonucleotide primers for PCR amplifications used in this study are listed in Table 4. These primers were synthesized by IDT (Integrated DNA Technologies, Coralville, IA).

**TABLE 4:** Oligonucleotide primers used in this study

Primers	Sequences (5′‐3′)	Note^a^
P_1_	GCAACAGAAAGAGGATTCTGA	Co‐RT‐PCR, *TDE1712*; [R]
P_2_	GAAATTAGGTATCTTTTGC	Co‐RT‐PCR, *TDE1713*; [F]
P_3_	CATAACATACCGATCGTAC	Co‐RT‐PCR, *TDE1713*; [R]
P_4_	GAATTAGGCTTTATCAGC	Co‐RT‐PCR, *TDE1714*; [F]
P_5_	GGTAACCGAAACTCCCGCAG	Co‐RT‐PCR, *TDE1714*; [R]
P_6_	GTTCAATCTTGCCGATAAG	Co‐RT‐PCR, *TDE1715*; [F]
P_7_	CTACACGCATTATTATGCG	Co‐RT‐PCR, *TDE1715*; [R]
P_8_	CGGCTCCTCCAAGAACCG	Co‐RT‐PCR, *TDE1716*; [F]
P_9_	CCCGGAGCGGCCGACATCAT	Co‐RT‐PCR, *TDE1716*; [R]
P_10_	GTTCAACATATGAGCAAAGC	Co‐RT‐PCR, *TDE1717*; [R]
P_11_	GAAAGATGCTCAAACACAAAAG	Co‐RT‐PCR, *TDE1002*; [R]
P_12_	CCAGATTCTCACACAGTCCG	Co‐RT‐PCR, *TDE1004*; [F]
P_13_	CGCCCGCTCGGTTAATACGT	Co‐RT‐PCR, *TDE1004*; [R]
P_14_	CATCCAACTGACGCTGTTGAG	Co‐RT‐PCR, *TDE1006*; [R]
P_15_	CCATTTCATCCGTGCGGATG	*TDE1716* 5′RACE outer primer
P_16_	GCAACACTGTTTAAAAACAT	*TDE1716* 5′RACE inner primer
P_17_	GCGCTCATGTTGTGATTAATG	*TDE1004* 5′RACE outer primer
P_18_	CATATTCCTACTCCTTTGG	*TDE1004* 5′RACE inner primer
P_19_	GAATTCTTCATTCTCCTTTTCCGTAAT	*TDE1716* promoter for pRS414; [F]
P_20_	GGATCCTTGTATACCTCTTTAAAAAA	*TDE1716* promoter for pRS414; [R]
P_21_	GAATCCGCAAAATTACGCTAATGAATG	*TDE1004* promoter for pRS414; [F]
P_22_	GGATCCTTAAAATTTAAGCTGTGC	*TDE1004* promoter for pRS414; [R]
P_23_	CCGTCCATTGTAGGCTTAC	qRT‐PCR, *dnaK*; [F]
P_24_	CCGTCAATGTCGATTCGTAC	qRT‐PCR, *dnaK*; [R]
P_25_	ATGAAAAAAACATTTATACTTG	qRT‐PCR, *flaA*; [F]
P_26_	CGAACAGAAAGAGGATTCTG	qRT‐PCR, *flaA*; [R]
P_27_	GGTGAAAATGTTGTTACCGGTTC	qRT‐PCR, *flaB1*; [F]
P_28_	GCTCCTATTTGCAGCATCGGC	qRT‐PCR, *flaB1*; *flaB2* replacement confirmation; [R]
P_29_	GGAGAAAATACCGTAACTGCTTC	qRT‐PCR, *flaB2*; [F]
P_30_	GGGTACCGATAGCGCGGTTAGC	qRT‐PCR, *flaB2*; [R]
P_31_	GGAGCACAACAAGGCGGAGAAG	qRT‐PCR, *flaB3*; [F]
P_32_	CCGTCATATGCCATTTCAAACC	qRT‐PCR, *flaB3*; *flaB2* replacement confirmation; [R]
P_33_	TACAACGGGACGGGACGGTCCC	5′portion for *flaB2* replacement; [F]
P_34_	GAATATTTTATATTTTTGTTCATGCGGCCGCATTCCTACTCCTTTGGTGATT	5′portion for *flaB2* replacement; [R]
P_35_	AATCACCAAAGGAGTAGGAATGCGGCCGCATGAACAAAAATATAAAATATTC	*ermB* for *flaB2* replacement; [F]
P_36_	TTATTTCCTCCCGTTAAATA	*ermB* for *flaB2* replacement; [R]
P_37_	TATTTAACGGGAGGAAATAATCATGACCTTGTTAAGGTAAG	3′portion for *flaB2* replacement; [F]
P_38_	TTAAACTACGATATTATGCTG	3′portion for *flaB2* replacement; [R]
P_39_	GCGGCCGCATGATTATCAATCACAACATG	*TDE1477* for *flaB2* replacement; [F]
P_40_	GCGGCCGCTTACCTTAAAAGAGACATTAC	*TDE1477* for *flaB2* replacement; [R]
P_41_	GCGGCCGCATGATTATTAATCACAATATG	*TDE1475* for *flaB2* replacement; [F]
P_42_	GCGGCCGCTTATTGAAGAAGCCTAACAAC	*TDE1475* for *flaB2* replacement; [R]
P_43_	TTATTTTTTAACTGCTTCATTC	*flaB2* replacement confirmation; [F]

^a^ Underlined sequences are engineered restriction cut sites for DNA cloning; [F] forward; [R] reverse.

### 4.2 PFs isolation

Isolation of the PFs was performed as previously described (Kurniyati et al., 2017). In brief, 500 ml of the mid‐logarithmic‐phase *T. denticola* cultures (~5 × 10^8^ cells/ml) were centrifuged at 5000× *g* for 20 min at 4°C. The cells were washed four times with phosphate‐buffered saline (PBS, pH 7.4) and once with T1 buffer (0.15 M Tris–HCl buffer, pH 6.8). The final pellets were resuspended in 30 ml of T1 buffer. Three milliliters of 10% Triton X‐100 were slowly added, mixed, and incubated for 1 h at room temperature. And then, 3 ml of 200 μg/ml of mutanolysin (Sigma‐Aldrich, St. Louis, MO) was added slowly, followed by the addition of 300 μl of T2 buffer (0.1 M Tris–HCl buffer, pH 6.8). The resultant mixture was first incubated for 2 h at room temperature and then at 4°C overnight. After the incubation, 600 μl of 0.1 M MgSO_4_ was added, followed by the addition of 600 μl of T2 buffer. The mixture was incubated for 5 min at room temperature and then centrifuged at 17,000× *g* for 15 min at 4°C. The supernatant containing PFs was collected and 2 ml of 20% PEG 8000 (Alfa Aesar, London, UK) in 1 M NaCl was added and then incubated for 30 min on ice. The resultant sample was centrifuged at 27,000× *g* for 30 min at 4°C. The pellet containing PFs was resuspended in alkaline solution (0.1 M KCl, 0.5 M sucrose, 0.1% Triton X‐100, 50 mM sodium bicarbonate, pH 11) and incubated for 1 h on ice. The PFs were finally collected by centrifugation at 80,000× *g* for 45 min at 4°C and washed once in 20 mM Tris–HCl buffer, pH 8. The PF pellet was resuspended in water and stored at 4°C for further analysis.

### 4.3 Gel electrophoresis

Two‐dimensional (2D) gel electrophoresis was carried out as previously described (Kurniyati et al., 2017). Equal amounts of purified PFs were resuspended in a rehydration buffer (5 M urea, 2 M thiourea, 2% CHAPS, 2% SB 3–10, 0.2% Bio‐Lyte 3/10 Ampholyte, 40 mM Tris, and 0.0002% Bromophenol Blue) and then subjected to separation using 7 cm long pH 5 → 8 linear IPG strips. The first dimension of isoelectric focusing (IEF) was performed using PROTEAN IEF (Bio‐Rad Laboratories, Hercules, CA), followed by equilibration according to the manufacturer's protocol. The second‐dimension separation was carried out using sodium‐dodecyl‐sulfate polyacrylamide‐gel electrophoresis (SDS‐PAGE) as described previously (Kurniyati et al., 2017). The resultant gels were subjected to Coomassie blue staining or immunoblotting analyses. The antibodies against *T. pallidum* FlaBs and *T. denticola* FlaA and DnaK are described in our previous publications (Kurniyati et al., 2017; Kurniyati & Li, 2016). The stoichiometry of each flagellar filament protein was analyzed using Image Lab software from Bio‐Rad (Bio‐Rad).

### 4.4 Mass spectrometry analysis of isolated PFs

Isolated PFs were treated with trypsin in an S‐Trap micro spin column (in solution digestion) (Zougman et al., 2014) and the resultant samples were subjected to nano‐LC‐ESI‐MS/MS analysis for protein identification which was carried out using an Orbitrap Fusion™ Tribrid™ (Thermo‐Fisher Scientific, San Jose, CA) mass spectrometer equipped with a nano‐spray Flex Ion Source, and coupled with a Dionex UltiMate 3000 RSLCnano system (Thermo, Sunnyvale, CA) as previously documented (Yang et al., 2018). The raw files with MS and MS/MS were subjected to database searches using Proteome Discoverer (PD) 2.5 software (Thermo Fisher Scientific, Bremen, Germany) with the Sequest HT algorithm. The PD 2.5 processing workflow containing an additional node of Minora Feature Detector for precursor ion‐based quantification was used for protein identification and relative quantitation of identified peptides and their modified forms. The database search was conducted against a *Treponema denticola* database downloaded from NCBI.

### 4.5 Measuring *T. denticola* growth rates

To measure the growth rates, 5 μl of the late‐log phase *T. denticola* cultures (10^8^ cells/ml) were inoculated into 5 ml of the TYGVS medium. *T. denticola* cells in the cultures were enumerated every 24 h using a Petroff Hausser counting chamber (Hausser Scientific, Horsham, PA). Each growth curve is representative of at least three independent cultures, and the results are represented as the mean of cell numbers ± standard error of the mean (SEM).

### 4.6 RNA preparations, RT‐PCR, qRT‐PCR, and RLM‐RACE

In brief, *T. denticola* cells were harvested at the mid‐logarithmic, late‐logarithmic, or stationary phase as indicated. Total RNA was extracted using TRI reagent (Sigma‐Aldrich), following the manufacturer's instructions. The resultant samples were treated with Turbo DNase I (Thermo Fisher Scientific, Waltham, MA) at 37°C for 2 h to eliminate residual genomic DNA contamination. The resultant RNA samples were re‐extracted using acid phenol‐chloroform (Ambion), precipitated in isopropanol, and washed once with 70% ethanol. The RNA pellets were resuspended in RNase‐free water. cDNA was generated from the purified RNA (1 μg) using SuperScript IV VILO cDNA synthesis kit (Thermo Fisher Scientific). qRT‐PCR was performed using iQ SYBR green supermix and a MyiQ thermal cycler (Bio‐Rad). The molecular chaperone DnaK gene (*dnaK*, *TDE0628*) was used as an internal control to normalize the qRT‐PCR data. The results were expressed as the normalized difference of the threshold cycle (ΔΔ*C*_*T*_) between the mid‐logarithmic‐phase cells and the other growth phase cells (the late log‐phase and the stationary‐phase). The primers used for qRT‐PCR are listed in Table 4. To determine the transcriptional start sites of flagellin genes, 5′RACE analysis was performed using the FirstChoice RLM‐RACE kit (Ambion), according to the manufacturer's protocol. Purified *T. denticola* RNA was reversed transcribed to cDNA with a 5′RACE adapter, followed by PCR amplification with primers listed in Table 4. The resultant PCR products were cloned into pGEM‐T easy vector (Promega, Madison, WI) and sequenced.

### 4.7 β‐Galactosidase activity assay

Fragments spanning from nucleotides −238 to +14 of *TDE1716* and −70 to −171 of *TDE1004* were PCR amplified with primers P_21_/P_22_ and P_23_/P_24_, respectively, generating fragments with engineered EcoRI and BamHI cut site at the 5′ and 3′ ends. The obtained fragments were in‐frame fused to the promoterless *lacZ* gene in the pRS414 plasmid (a gift from R. Breaker, Yale University), creating two transcription report vectors, *P*_*flaA*_ and *P*_*flaB2*_. The *P*_*flaB1*_ vector was constructed in our previous study (Kurniyati et al., 2019). The plasmids were transformed into *E. coli* DH5α. The β‐galactosidase activity was measured and expressed as the average Miller units of triplicate samples from three independent experiments.

### 4.8 Constructions of *T. denticola* flagellin gene mutants

The *flaA* and three *flaB* genes were previously deleted through DNA allelic exchange (Kurniyati et al., 2017). The resultant four mutants were named: *ΔflaA*, *ΔflaB1*, *ΔflaB2*, and *ΔflaB3*. To determine if three *flaB* genes have an overlap function, we constructed two vectors to in‐frame replace *flaB2* (*TDE1004*) with either *flaB1* (TDE1477) or *flaB3* (TDE1475). These two vectors were constructed by two‐step PCR alongside DNA cloning as previously documented (Bian et al., 2012). In brief, the *flaB2* upstream flanking region (*B2UR*), the *ermB* cassette (*ermB*), and the *flaB2* downstream region (*B2DR*) were PCR amplified with primers P_33_/P_34_, P_35_/P_36_ and P_37_/P_38_, respectively. And then, *B2DR* and *ermB* were PCR ligated with P_35_/P_38_, generating the fragment of *ermB‐B2DR* which was then PCR ligated to *B2UR* with primers P_33_/P_38__._ The final PCR fragment, *B2UR‐ermB‐B2DR*, was cloned into the pMD19 T‐vector (Takara Bio USA, Inc, Mountain View, CA). Of note, a NotI cut site was engineered between *B2UR* and *ermB* for subcloning. The intact *flaB1* and *flaB3* genes were PCR amplified with primers P_39_/P_40_ and P_41_/P_42_, respectively, and then cloned into the pGEM‐T easy vector (Promega). The cloned *flaB1* and *flaB3* genes were then released by NotI and subcloned into *B2UR‐ermB‐B2DR*, generating two vectors, one with *B2UR‐flaB1‐ermB‐B2DR* and the other with *B2UR‐flaB3‐ermB‐B2DR*. To replace *flaB2* with *flaB1* or *flaB3*, these two vectors were transformed into *T. denticola* wild‐type competent cells via electroporation as previously described (Kurniyati & Li, 2021). The obtained two mutants, *ΔflaB2/B1* and *ΔflaB2/B3*, were confirmed using PCR and immunoblotting.

### 4.9 Bacterial swimming plate assay and motion tracking analysis

A swimming plate assay of *T. denticola* was performed as previously described (Kurniyati et al., 2013). In brief, 3 μl of cultures (10^9^ cells/ml) were inoculated onto 0.35% agarose containing the TYGVS medium diluted 1:1 with PBS. The plates were incubated anaerobically at 37°C for 3–5 days to allow the cells to swim out. The diameters of the swimming rings were measured in millimeters. As a negative control, a previously constructed *T. denticola* non‐motile mutant, *Δtap1*, was included to determine the initial inoculum size (Limberger et al., 1999). The average diameters of each individual strains were calculated from three independent plates; and the results are represented as the mean of diameters ± standard error of the mean (SEM). The velocity of bacterial cells was measured using a computer‐based bacterial tracking system, as previously described (Kurniyati et al., 2019). In brief, 100 μl of mid‐logarithmic‐phase *T. denticola* cultures was first diluted (1:1) in TYGVS medium and then 10 μl of diluted cultures were mixed with an equal volume of 2% methylcellulose with a viscosity of 4000 cp (MC_4000_). *T. denticola* cells were videotaped and tracked using a computer‐based bacterial tracking system alongside the software Volocity (Improvision Inc., Coventry, United Kingdom), as described before. For each bacterial strain, at least 24 cells were recorded for up to 30 sec. The average cell swimming velocities (μm/s) of tracked cells were calculated. The data were statistically analyzed by one‐way ANOVA followed by Tukey's multiple comparison at *p* < .01.

### 4.10 TEM of PFs

5 μl of the purified PF were applied to Formvar–carbon copper grids (Electron Microscopy Sciences, Hatfield, PA) and negative stained with 1% uranyl acetate for 1 min (pH 4.2). The samples were subjected to a JEOL JEM‐1400plus transmission electron microscope (TEM) at an acceleration voltage of 120.0 kV. Flagellar helix pitch and diameter were measured using Fiji software.

### 4.11 Cryo‐ET sample preparation, data collection, and image processing

The frozen‐hydrated specimens of *T. denticola* were prepared as previously described (Kurniyati et al., 2017; Kurniyati et al., 2019). In brief, *T. denticola* cultures were mixed with 10 nm colloidal gold solutions and then deposited onto a freshly glow‐discharged, holey carbon grid for about one minute. The grids were blotted with a small piece of filter paper for ~4 s and then rapidly plunged in liquid ethane using a gravity‐driven plunger apparatus. The frozen‐hydrated specimens of *T. denticola* were transferred to a 300 kV electron microscope (Krios, Thermo Fisher Scientific) or a 200 kV electron microscope (Glacios, Thermo Fisher Scientific) that is equipped with a field emission gun and a direct detection detector (K2, Gatan). To generate three‐dimensional (3D) reconstructions of whole bacterial cells, tomographic package SerialEM was used to acquire multiple tilt series along the cell. The pixel size at the specimen level is 5.1 Å. All tilt series were collected in the low‐dose mode with ~8 μm defocus. A total dose of 60 e^−^/Å^2^ is distributed among 35 tilt images covering angles from −51° to +51° at tilt steps of 3°. The tilt series were aligned and reconstructed by IMOD. Multiple reconstructions from different segments of the same cell were integrated into a whole‐cell reconstruction. In total, 187 tomograms were generated to build whole‐cell reconstructions from WT (12), *ΔflaA* (6), *ΔflaB1*(7), *ΔflaB2* (8), *and ΔflaB3* (4) cells, respectively.

### 4.12 Statistics analyses

For quantitative experiments (e.g., swimming plate assay, tracking analysis, and measurement of PFs length, diameter, and helicity), multiple samples were included and at least three independent experiments were conducted. The results are expressed as mean ± standard errors of mean (SEM). Statistical significance was analyzed by one‐way ANOVA followed by Tukey's multiple comparison at *p* < .01.

## AUTHOR CONTRIBUTIONS

KK and YC performed the experiments and analyzed results. JL and CL designed the study and wrote the manuscript. All authors read and approved the manuscript.

## CONFLICT OF INTEREST

The authors declare no conflict of interest.

## ETHICS STATEMENT

All animal experimentation was carried out in strict accordance with the recommendations in the Guide for the Care and Use of Laboratory Animals of the National Institutes of Health. The protocol for animal studies was approved by the Institutional Animal Care and Use Committee (permit number: AD10001778) of Virginia Commonwealth University.

## Supporting information


Video 1

Video 2

Video 3

Video 4

Video 5

Appendix S1

## Data Availability

The data that support the findings of this study are available from the corresponding author upon reasonable request.
