# Supplementary material for: Transcriptional and functional characterizations of multiple flagellin genes in spirochetes
Source: Mol Microbiol. 2022 Jul 18;118(3):175–90. doi: 10.1111/mmi.14959 (PMC9481697; doi:10.1111/mmi.14959)
Supplement: Supplementary file 6 — Appendix S1 [file MMI-118-175-s004.pdf]

Transcriptional and functional characterizations of multiple flagellin genes in spirochetes

Kurni Kurniyati[1], Yunjie Chang[3,4], Jun Liu[3,4*], and Chunhao Li[1,2*]

[1]*Philips Institute for Oral Health Research, School of Dentistry;* [2]*Department of Microbiology and Immunology, School of Medicine, Virginia Commonwealth University, Richmond, VA, USA*

[3]*Microbial Sciences Institute, Yale University, West Haven, CT, USA*

[4]*Department of Microbial Pathogenesis, Yale School of Medicine, New Haven, CT, USA*

Running title: the role of multiple flagellins in spirochetes

Keywords (Spirochetes, *Treponema*, Flagellin, Motility, and Sigma factors)

*Corresponding author. Mailing address: Philips Institute for Oral Health Research, School of Dentistry, Virginia Commonwealth University, Richmond, VA 23298, USA

Electronic mail address: cli5@vcu.edu; phone: (804) 628-4401

Table S1. Proteins identified in the purified PFs of *T. denticola* by using LC-MS/MS analysis.

	A	B	C	D	E	F	G	H	I	J	K	L	M	N	O	P	Q	R
1	Accession	Description	Coverage [%	# Peptides	# PSMs	# Unique Pe	# AAs	MW [kDa]	calc. pI	Score Sequ	# Peptides (	Pfam IDs	Entrez Gene	Gene ID	Gene Symb	Abundance	Abundance	Found in Sa
2	WP_002668950.1	flagellin [Treponema denticola]	82	16	1730	4	285	30.9	5.49	9052.74	16	Pf00669, P	2741590	A0A0E2E7U	FlaB3	100	1.01E+11	High
3	WP_002681434.1	MSP porin [Treponema denticola]	78	27	867	27	543	58.2	6.83	3773.16	27	Pf02707, P	2740151	F7IWF6; Q5	Msp	100	4.47E+10	High
4	WP_002670927.1	flagellar filament outer layer protein FlaA [Treponema denticola]	73	30	1259	12	349	39.3	5.53	4774.28	30	Pf04620	2740884	A0A0E2EGF	FlaA	100	2.9E+11	High
5	WP_002668952.1	flagellin [Treponema denticola]	67	16	973	4	286	31.3	6.13	5035.61	16	Pf00669, P	2741592	A0A0E2E87	FlaB1	100	5.25E+10	High
6	WP_002680830.1	flagellar hook protein FlgE [Treponema denticola]	89	21	214	21	463	49.5	4.98	921.55	21	Pf00460, P	2740735	F7IUZ4; Q7	FlgE	100	6.79E+09	High
7	WP_002670454.1	flagellin [Treponema denticola]	64	15	852	11	286	31.5	7.06	3996.83	15	Pf00669, P	2740984	A0A0E2E2C	FlaB2	100	6.13E+10	High
8	WP_002674261.1	HEAT repeat domain-containing protein [Treponema denticola]	59	12	459	12	234	26.2	5.54	2038.91	12	Pf13646	2739958	A0A0E2EM		100	2.6E+10	High
9	WP_002680269.1	flagellar hook-associated protein FlgK [Treponema denticola]	45	19	67	19	623	69.5	4.94	245.73	19			WP_00268	FlgK	100	9E+08	High
10	WP_002680271.1	flagellar hook-associated protein 3 [Treponema denticola]	48	13	32	13	415	45.9	5.25	117.01	13	Pf00669, P	2739962	Q73K69; tc	FliD	100	6.34E+08	High
11	WP_002668466.1	flagellar M-ring protein FliF [Treponema denticola]	27	12	43	12	567	64.8	5.69	133.4	12	Pf01514, P		E9S3X0; W	FliF	100	7.52E+08	High
12	WP_002670463.1	flagellar basal-body rod protein FlgG [Treponema denticola]	56	8	23	8	264	28.8	5.72	86.94	8	Pf00460, P	2740987	A0A0E2E3N	FlgG-2	100	3.58E+08	High
13	WP_002666989.1	flagellar basal body-associated FliL family protein [Treponema den	22	4	21	4	181	20.6	5.99	100.36	4	Pf03748	2740731	A0A0E2ECF	FliL	100	3.25E+08	High
14	WP_002671871.1	flagellar basal-body rod protein FlgF [Treponema denticola]	34	6	10	6	270	30.2	4.89	42.03	6	Pf00460, P	2740986	A0A0E2E1F	FlgG-1	100	90495112	High
15	WP_002671661.1	flagellar basal body rod protein FlgC [Treponema denticola]	34	3	15	3	151	16.6	7.25	50.44	3	Pf00460, P	2740107	A0A0E2E8C	FlgC	100	20458079	High
16	WP_002668460.1	flagellar basal body rod protein FlgB [Treponema denticola]	21	3	3	3	139	15.8	7.5	11.55	3	Pf00460	2740106	E9S3W7; M	FlgB	100	2657346	High

Table S2. Characterization of *T. denticola* wild type and four flagellar filament gene deletion mutants by using cryo-electron tomography.

	Cell length (μm)	PFs length (μm)	PFs length (old pole, μm)	PFs diameter (nm)
**WT**	Cell 1 (7.0)	4.4<br>4.7	6.5<br>6.3	19.5 ± 1.7<br>14.2 ± 1.4
	Cell 2 (7.6)	3.0<br>3.1	7.5<br>7.5	
ΔflaA	Cell 1 (11.0)	3.0<br>3.5	8.0<br>8.2<br>7.0	13.9 ± 1.5
	Cell 2 (11.5)	3.6<br>4.0	5.0<br>12.0<br>12.0	
ΔflaB1	Cell1 (5.5)	1.5<br>2.2<br>2.5	3.9	19.9 ± 2.1<br>13.0 ± 1.1
	Cell 2 (7.0)	2.0<br>3.0	4.0<br>5.0	
ΔflaB2	Cell 1 (6.0)	1.2<br>2.0	1.5<br>3.0	21.1 ± 1.4<br>13.6 ± 1.6
	Cell 2 (7.0)	3.0	4.3<br>6.4	
ΔflaB3	Cell 1 (7.0)	2.4	4.4	20.6 ± 2.0<br>13.4 ± 1.6
	Cell 2 (6.5)	3.0	5.0	

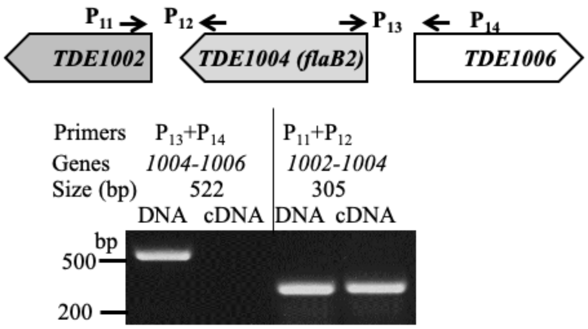

Figure S1. Co-RT-PCR analysis of *TDE1002-TDE1006* genes. This experiment was performed as previously documented (Kurniyati *et al.*, 2019). Two pairs of primers that bridge *flaB2* and its flanking genes were designed and used for co-RT-PCR. For each co-RT-PCR reaction, a parallel PCR reaction was performed and used as a positive control. The resultant co-RT-PCR and PCR products were detected in 2% agarose gel electrophoresis.

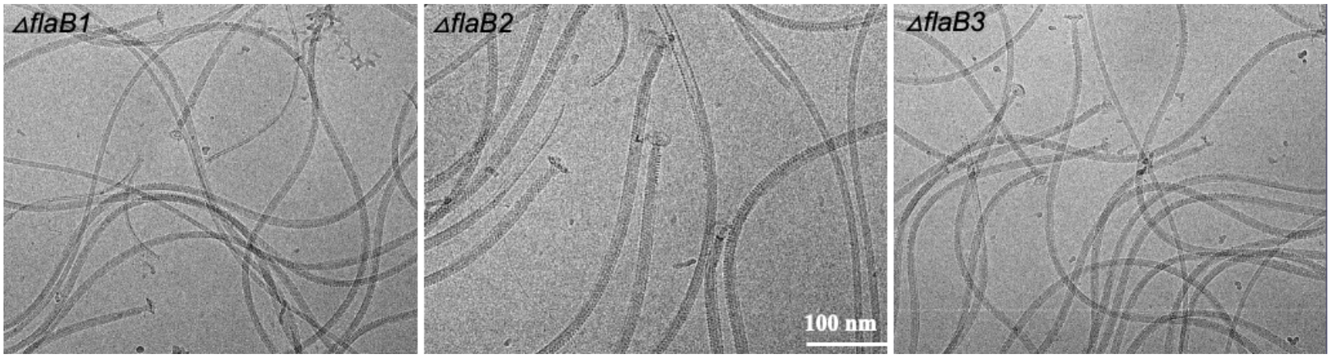

Figure S2. Representative cryo-electron microscopic images of PFs isolated from *ΔflaB1*, *ΔflaB2*, and *ΔflaB3* mutants.

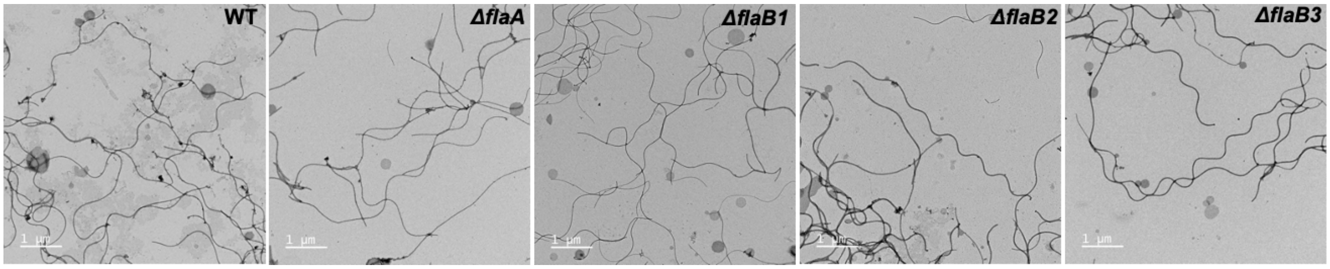

Figure S3. Representative TEM images of PFs isolated from *T. denticola* wild type (WT) and four flagellar filament gene deletion mutants.

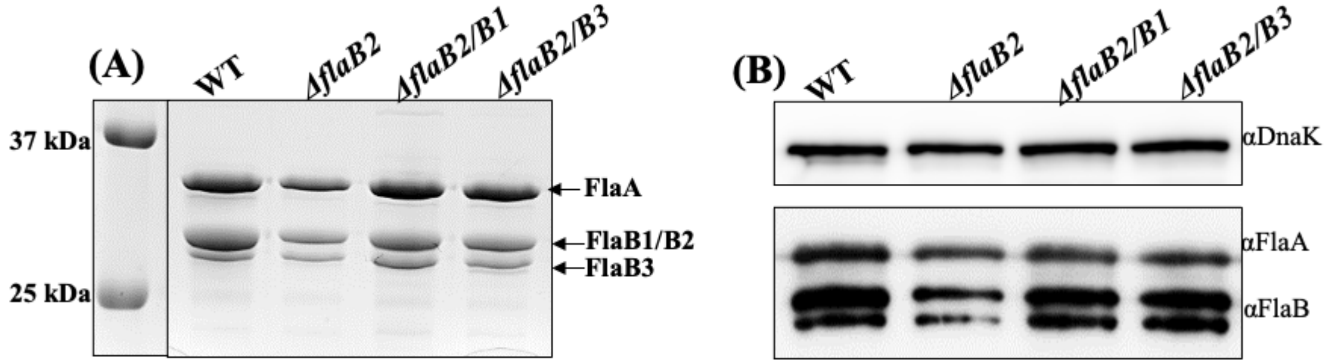

Figure S4. Characterizations of two *flaB2* gene replaced mutants: *ΔflaB2/B1* and *ΔflaB2/B2*. (A) SDS-PAGE analysis of PFs isolated from WT, *ΔflaB2, ΔflaB2/B1*, and *ΔflaB2/B3* mutants. (B) Western-blot analysis of isolated PFs. For the immunoblotting, antibodies against *T. denticola* DnaK (αDnaK), FlaA (αFlaA), and *T. pallidum* FlaB (αFlaB) were used.

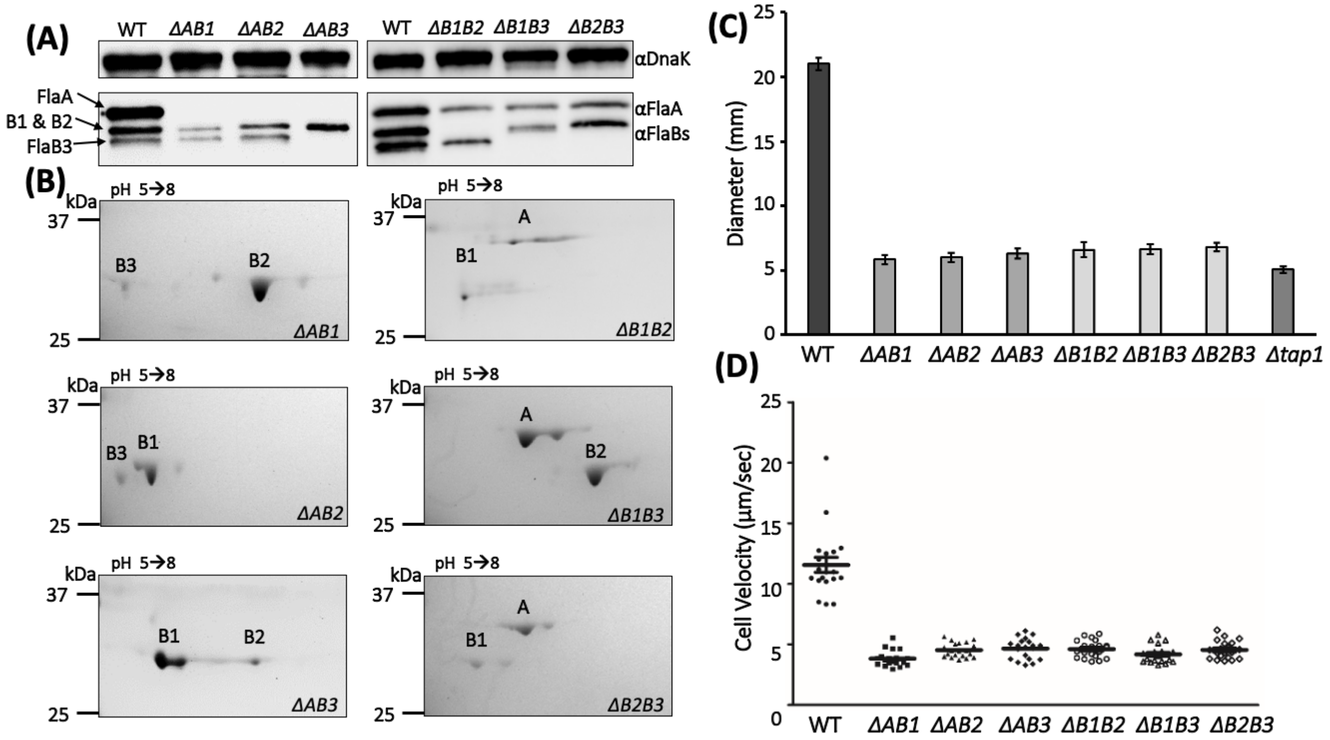

Figure S5. Characterizations of six double deletion mutants of *T. denticola*. **(A)** Whole cell lysate immunoblotting analysis of WT and six flagellar filament gene double deletion mutants. The blots were probed with antibodies against *T. denticola* DnaK (αDnaK), FlaA (αFlaA), and *T. pallidum* FlaB (αFlaB), respectively. DnaK was used as a loading control. **(B)** 2D gel electrophoresis of six double mutants, followed by immunoblotting with antibodies against *T. denticola* FlaA (αFlaA) and *T. pallidum* FlaB (αFlaB). **(C)** Swimming plate assay. This assay was carried out on 0.35% agarose plates containing the TYGVS medium diluted 1:1 with PBS. The plates were incubated anaerobically at 37°C for 3 days to allow the cells to swim out. *Δtap1*, a previously constructed non-motile mutant (Limberger *et al.*, 1999), was used as a control to determine the initial inoculum sizes. The sizes of swimming rings from five different plates were measured and averaged. **(D)** Cell tracking analysis. *T. denticola* cells were tracked in the presence of 1% methylcellulose, as previously described. The results are expressed as the mean of μm/s ± standard errors of mean (SEM). WT: wild type; *ΔAB1*: *ΔflaAflaB1*; *ΔAB2*: *ΔflaAflaB2*; *ΔAB3*: *ΔflaAflaB3*; *ΔB1B2*: *ΔflaB1flaB2*; *ΔB1B3*: *ΔflaB1flaB3*; and *ΔB2B3*: *ΔflaB2flaB3*.
